# Weight Bearing Over-ground Stepping in an Exoskeleton with Non-invasive Spinal Cord Neuromodulation after Motor Complete Paraplegia

**DOI:** 10.3389/fnins.2017.00333

**Published:** 2017-06-08

**Authors:** Parag Gad, Yury Gerasimenko, Sharon Zdunowski, Amanda Turner, Dimitry Sayenko, Daniel C. Lu, V. Reggie Edgerton

**Affiliations:** ^1^Department of Integrative Biology and Physiology, University of California, Los AngelesLos Angeles, CA, United States; ^2^Pavlov Institute of PhysiologySt. Petersburg, Russia; ^3^Department of Neurosurgery, University of California, Los AngelesLos Angeles, CA, United States; ^4^Brain Research Institute, University of California, Los AngelesLos Angeles, CA, United States; ^5^Department of Neurobiology, University of California, Los AngelesLos Angeles, CA, United States; ^6^Institut Guttmann, Hospital de Neurorehabilitació, Institut Universitari adscrit a la Universitat Autònoma de BarcelonaBarcelona, Spain

**Keywords:** spinal cord injury, exoskeleton, spinal cord stimulation, non-invasive neuromodulation, neural prostheses for locomotion, locomotion rehabilitation

## Abstract

We asked whether coordinated voluntary movement of the lower limbs could be regained in an individual having been completely paralyzed (>4 year) and completely absent of vision (>15 year) using two novel strategies—transcutaneous electrical spinal cord stimulation at selected sites over the spine as well as pharmacological neuromodulation by buspirone. We also asked whether these neuromodulatory strategies could facilitate stepping assisted by an exoskeleton (EKSO, EKSO Bionics, CA) that is designed so that the subject can voluntarily complement the work being performed by the exoskeleton. We found that spinal cord stimulation and drug enhanced the level of effort that the subject could generate while stepping in the exoskeleton. In addition, stimulation improved the coordination patterns of the lower limb muscles resulting in a more continuous, smooth stepping motion in the exoskeleton along with changes in autonomic functions including cardiovascular and thermoregulation. Based on these data from this case study it appears that there is considerable potential for positive synergistic effects after complete paralysis by combining the over-ground step training in an exoskeleton, combined with transcutaneous electrical spinal cord stimulation either without or with pharmacological modulation.

## Introduction

The mammalian spinal cord is capable of generating locomotor output independent of any input from the brain using locomotor related neuronal circuitries. After complete mid-thoracic transection of the spinal cord, paralyzed cats can stand, and step when appropriate proprioceptive input is provided to the lumbosacral networks that contain the pattern generator circuitry (Rossignol et al., 2007; Rossignol and Frigon, 2011). The animals can learn to fully support their hindquarters, and to step at a range of speeds and loads (de Leon et al., 1999a,b). Adult paralyzed rats can relearn to step with a combination of neuromodulatory strategies including locomotor training, pharmacological intervention, and epidural stimulation (Courtine et al., 2009; Musienko et al., 2012; Gad et al., 2013, 2015). Paralyzed rats can step forward, sideways, backwards as well as climb stairs voluntarily (Shah et al., 2012). Recently we have suggested a novel neuromodulation strategy of motor control using non-invasive transcutaneous spinal cord stimulation combined with administering a monoaminergic agent. We have demonstrated that transcutaneous spinal cord stimulation at lumbosacral segments can induce coordinated stepping like movements in paralyzed individuals when their lower limbs are suspended in a gravity neutral device (Gorodnichev et al., 2012; Gerasimenko Y. et al., 2015). Also this strategy has enabled voluntary control of stepping-like motions in 5/5 individuals with chronic complete motor paralysis (Gerasimenko Y. P. et al., 2015).

Here, we use this non-invasive stimulation technology, painless cutaneous enabling motor control (pcEmc) to determine the feasibility of re-establishing functional brain spinal cord connectivity that enables a subject with complete motor paralysis to move upon volitional intent and perform work that can assist a robotic exoskeletal device in generating over-ground stepping.

The use of robotic-like devices to improve locomotion in patients with paralysis has been tested with mixed results (Ferris et al., 2005; Esquenazi et al., 2012; Strausser et al., 2012; McDaid et al., 2013). In experimental studies in animals, we developed an assist-as-needed (AAN) robotic treadmill device with arms to move the legs in a step-like trajectory so that the mice could be trained with an allowable variation in window diameter and temporal pattern controlled with a graded force field (Cai et al., 2006). Several Exoskeleton devices have been developed to allow human subjects to step over-ground with varying efforts from the subject including some that are approved by FDA such as the ReWalk, Indego, EKSO Bionics and others that are still experimental (Esquenazi et al., 2012; del-Ama et al., 2012). Some of the experimental units include a brain computer interface (Lebedev and Nicolelis, 2011; Onose et al., 2016) or muscle stimulators (del-Ama et al., 2014), however, each of these units bypass the automaticity that is intrinsic to the spinal networks and do not allow voluntary effort from the subjects and the inter-step variability to allow the spinal networks to relearn stepping (Ziegler et al., 2010).

EKSO Bionics is a battery powered wearable bionic suit with motors at the hips and knees which enables individuals with lower extremity motor impairment to stand and voluntarily step over-ground with weight-bearing and alternating gait. The EKSO GT robotic exoskeleton is a class I medical device (United States FDA) which provides functional rehabilitation in the form of over-ground weight bearing stepping in subjects with spinal cord injuries (complete and incomplete) and stroke. The device works in two modes namely the “max assist” and “variable assist.” In max assist, the pilot (patient) has to initiate a step by unweighting one leg which triggers the motors on the EKSO to move the entire limb in a step like trajectory. In this mode, 100% assistance is offered during the entire step cycle. However, the variable assist mode actively allows the subject to voluntarily assist, even when the subject exerts minimal voluntary influence on the robot.

Thus, the objective of the study was to test the combinatorial effects of non-invasive electrical spinal cord stimulation, pharmacological neuromodulation with a robotic device that allows one to voluntarily assist the robot during over-ground stepping. We hypothesized that tonic pcEmc can modulate the paralyzed spinal networks so that the subject can voluntarily engage these networks to assist stepping, as observed in spinal mice, when training in the EKSO. The present results demonstrate that locomotor spinal networks can be neuromodulated with pcEmc to physiological states, similar to that observed in paralyzed mice with epidural stimulation that enables sensory input to serve as a source of neural control to generate stepping. Based on our previous observation with epidural stimulation we also hypothesized that the plasticity occurring in the spinal cord would re-enable improved voluntary control of lower limbs other than during stepping, as well as cardio-vascular function.

## Methods

### Clinical status

**Figure 1:**
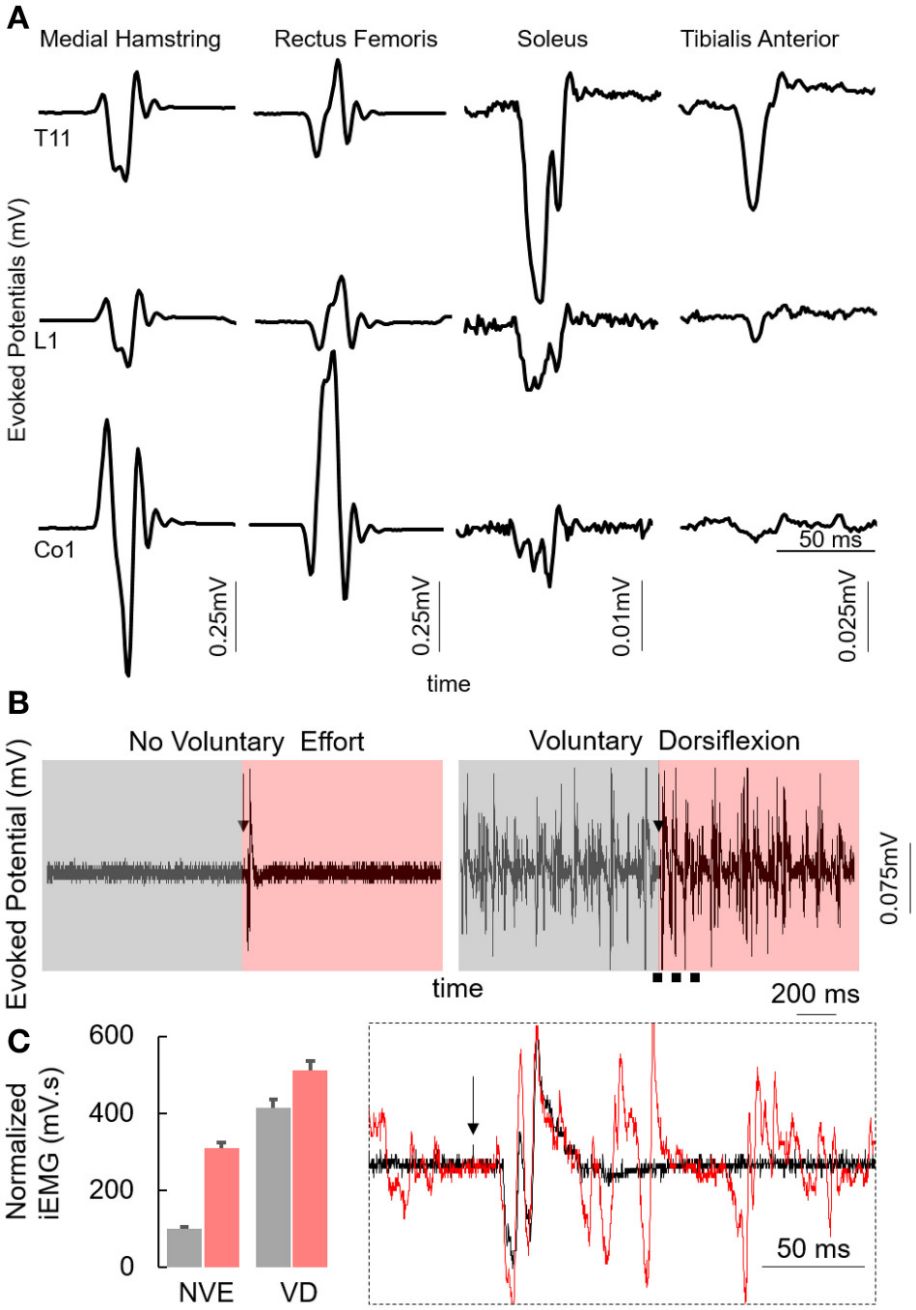
**(A)** Average motor evoked potentials (*n* = 5) at 200 mA while stimulating the T11, L1, and Co1 vertebral levels with the subject in supine position from the multiple lower limb muscles. **(B)** Average motor evoked potentials (*n* = 3) from the MG muscles with the subject in supine position at T11 (110 mA) vertebral level during control conditions (No Voluntary Effort, NVE) or with the subjects voluntarily attempting to maximally dorsiflex (Voluntary Dorsiflexion, VD). Box inset: zoomed in view during NVE and voluntary dorsiflexion 1 s before the stim pulse (gray) and 1 s after the stim pulse (red). Note the black arrow marks the stimulation pulse. **(C)** Normalized mean+SD integrated EMG for the duration highlighted in **(B)**. Inset represents the zoomed in view of the region marked by black dotted line. Each of these data were collected at PreStim time point.

The subject was an adult between the age of 35 and 40 years at the time of the study. The subject was diagnosed with a detached retina at the age between 20 and 25 years leading to complete blindness. In 2010 the subject fell from a 2nd story balcony hitting a concrete floor causing a spinal cord injury at T9 and L1 vertebral levels. The subject was initially (immediately after the injury), 24 months post injury and immediately before the study was initiated, was diagnosed as American Spinal Injury Association Impairment Scale (AIS) A (no sensation or movement below the level of injury) motor complete injury and had no motor function of trunk or leg muscles and used a suprapubic catheter to enable bladder emptying. Despite being diagnosed as AIS A, the spinal locomotor circuitry in the lumbosacral spinal cord was active and responsive to stimulation (Figure 1A) that was varied based on the site of stimulation with the generation of late responses (latency > 100 ms) and increased muscle activity while voluntarily attempting dorsiflexion or plantarflexion (Figures 1B,C). The subject had been training (inconsistently) with the EKSO Bionics suit for over 24 months and had completed ~180,000 steps prior to the experiment. The subject used crutches while stepping in the EKSO during the entire period of the study. The subject signed an informed consent form which was approved by the Institutional Review Board (IRB) at the University of California, Los Angeles (UCLA).

### Experimental procedures

#### Training: walking in the EKSO bionics

The study was divided into four phases, baseline, stim only, drug only, and drug+stim (1 week each). EMG and EKSO robot data were collected at the end of each phase of the study. Further, self-scored data from the subject were collected everyday (see below for details).

To establish and define a functional baseline the subject walked in the EKSO for a period of 4 weeks without pcEmc in variable assist mode for 1 h/day, 5 days/week. Each 1-h session was divided into blocks of 15 min with 5 min break in between. At the end of the 4-week period, EKSO training with neuromodulation was initiated. Neuromodulation consisted of 1 week of pcEmc only followed by 1 week of pharmacological enabling motor control (fEmc) followed by 1 week of pcEmc and fEmc. Each day of training consisted of 5–10 min warm up block, followed by three 20 min blocks with or without pcEmc based on the phase of the study. Blood pressure and heart rate readings were recorded at the end of each block.

### Neuromodulation parameters

Non-invasive spinal cord stimulation at T11 and/or Co1 was administered using a specific stimulation waveform that minimizes discomfort, even when used at energies required to transcutaneously reach the spinal cord (Gerasimenko Y. P. et al., 2015). pcEmc was delivered using a 2.5 cm diameter electrode (Lead Lok, Sandpoint, United States) placed midline on the skin between spinous processes T11–T12 (simply T11) or over Co1 as a cathode and two 5.0 × 10.2 cm^2^ rectangular plates made of conductive plastic (Ambu, Ballerup, Germany) placed symmetrically on the skin over the iliac crests as anodes. pcEmc was delivered at Thorasic T11 – 30 Hz and/or Coccyx bone segment (Co1) – 5 Hz, the intensities of stimulation were determined based on optimum efficacy and feedback from the subject. fEmc was administered with 10 mg tablets twice a day (prescribed by the MD on the study).

### Testing

Baseline (pre-train) data were collected after 4 weeks of training. An external pressure sensor (FSR 1 sq. inch) was attached to the right heel at the same site as the internal pressure sensor on the EKSO. This FSR pressure sensor was connected to the EMG system (Delsys System) allowing us to synchronize the data generated by the EKSO and the EMG system. At the end of each phase, ability to step in the EKSO was tested both with and without pcEmc. Data were synchronized, band pass filtered and analyzed using custom scripts written in Matlab (Mathworks). 30 consecutive steps when the subject was stepping was chosen to perform the data analysis including calculating mean assistance data from the EKSO, mean integrated EMG from proximal, and distal muscles. Similarly, ability to flex specific joints was tested at the end of each phase with and without pcEmc.

### EKSO bionics

The EKSO device can work in two modes namely the “max assist” and “variable assist.” In max assist, the pilot (patient) has to initiate a step by unweighting one leg which triggers the motors on the EKSO to move the entire limb in a step like trajectory. In this mode, 100% assistance is offered during the entire step cycle. However, in the variable assist mode actively allows the subject to voluntarily assist, even when the subject voluntarily exerts minimal influence on the robot.

During training, the EKSO device was operated in variable assist mode enabling the subject to generate voluntary effort to move forward and maintain a predetermined trajectory. During the course of each step, if the subject's voluntary effort was not sufficient to maintain the trajectory, the onboard computer provided the required assistance to complete the step as planned. The EKSO device contained multiple position sensors (hip, knee, torso) that prevented the subject from falling and recorded 74 parameters (sampled at 500 Hz) including the assistance provided and current drawn by the motors which are directly related to the subjects' efforts to enable weight bearing stepping.

### Kinematics and EMG recordings

Bipolar surface electrodes were placed bilaterally on the soleus, medial gastrocnemius (MG), tibialis anterior (TA), medial hamstring (HM), and vastus lateralis (VL) muscles as described previously (Gerasimenko Y. P. et al., 2015). Electromyography (EMG) signals were amplified differentially (bandwidth of 10 Hz to 10 kHz) and acquired at 10 KHz using a 16-channel hard-wired A/D board and customized LabVIEW software (National Instruments, Austin, TX) acquisition program. To minimize artifacts from the stimulation, the EMG signals were passed through a band-pass filter using a six-order band-pass Butterworth filter (30–1,000 Hz). The filtered EMG signals were analyzed off-line to compute the amplitude, duration, and timing of individual bursts. Angular displacements at hip and knee joints in both legs were recorded with goniometers.

### Self-scoring by subject

The subject self-scored on various parameters including (1) muscle tone during stepping, (2) sensation during stepping, (3) sensation in legs every morning, (4) body perspiration during stepping, (5) hand to leg coordination: This scores the perception of the subject's ability to move the upper and lower extremity as a synchronous in a smooth and synchronous manner rather than indiscreet interrupted components.

## Results

### Active vs. passive stepping

**Figure 2:**
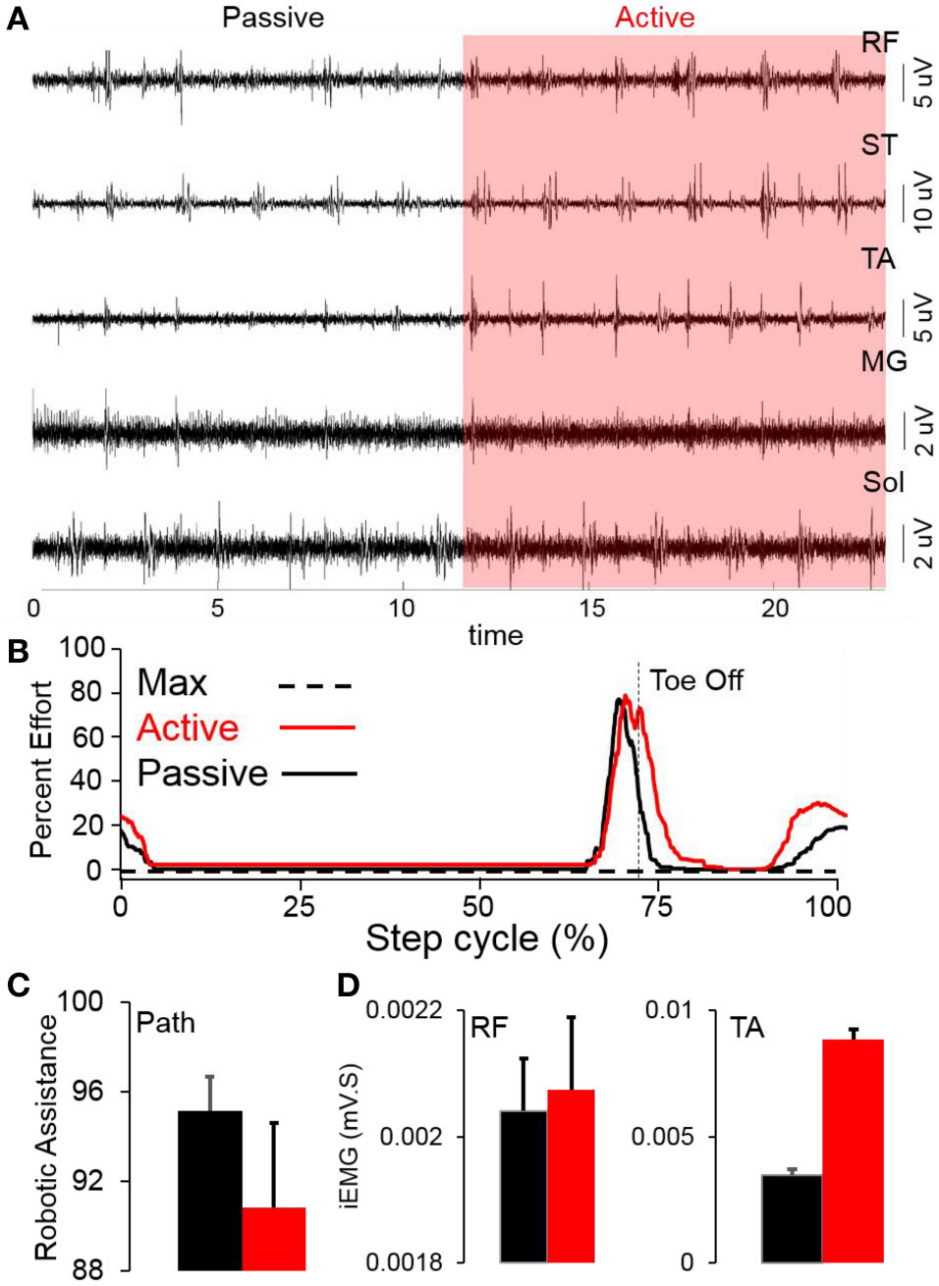
**(A)** Raw EMG from multiple leg muscles while stepping in the EKSO without (passive) and with (red highlight) voluntary effort (active) **(B)** Average percent effort (*n* = 30 steps) during either active or passive stepping during a normalized step cycle. **(C)** Mean+SD (*n* = 30 steps) robotic work during stepping in the EKSO (%). **(D)** Mean+SD (*n* = 30 steps) integrated EMG in rectus femoris (RF) and tibialis anterior (TA) while stepping in the EKSO in active (red) or passive (black) modes.

**Figure 3:**
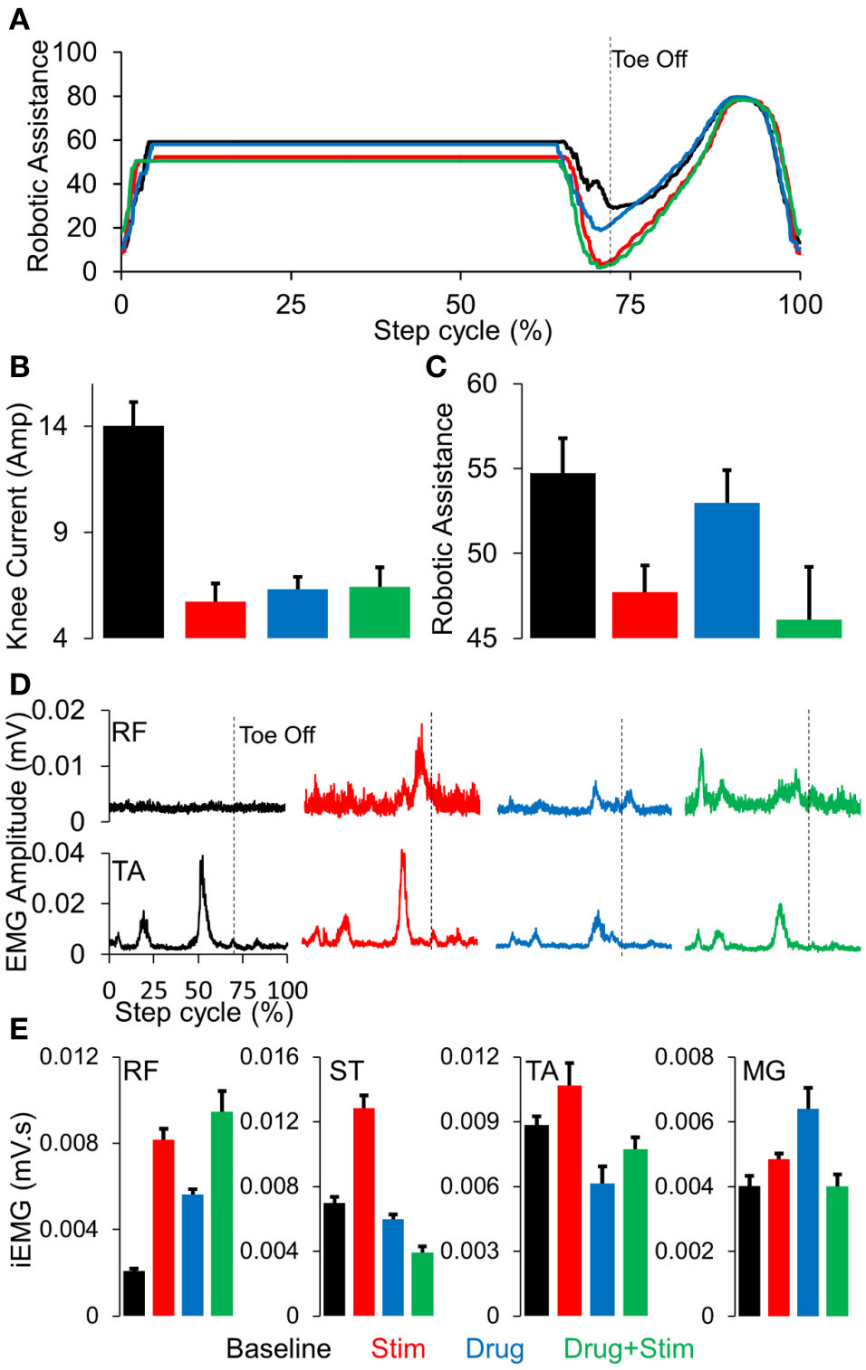
**(A)** Average robotic assistance (*n* = 30 steps) during a complete normalized step cycle during baseline (black), stim (red), drug (blue), and stim+drug (green) phases while stepping in active mode. **(B)** mean+SD (*n* = 30 steps) knee current in the conditions mentioned above. **(C)** mean+SD (*n* = 30 steps) robotic assistance in the conditions mentioned above. **(D)** Average (*n* = 30 steps) linear envelope for the RF and TA muscles while stepping in the EKSO, **(E)** Mean+SD (*n* = 30 steps) integrated EMG while stepping in the EKSO in active mode for the conditions mentioned above. RF, Rectus Femoris; ST, Semitendinosus; TA, Tibialis Anterior; MG, Medial Gastrocnemius.

Stepping passively (in variable assist mode) or in max assist in the EKSO resulted in minimal activation of lower extremity muscles and autonomic function (perspiration etc) over the course of the entire period of testing each day as well as weeks of training. Active voluntary effort during stepping resulted in increased EMG activity especially in proximal muscles, heart rate and blood pressure along with decrease in assistance provided by the EKSO. The decreased assistance was accompanied by increased EMG activity in proximal and distal muscles (Figure 2). Further, in the presence of stimulation, active stepping resulted in less assistance and higher levels of EMG activity (Figure 3A). The changing mean assistance, motor current and activation patterns of muscles varied with the site of stimulation (Figures 3B,C). However, the most effective pattern of stimulation (least mean assistance and maximum EMG activity) was observed during a combination of T11 and Co1 stimulation (Figures 3D,E). fEmc alone, on the other hand, required a higher level of assistance by the EKSO compared to pcEmc and voluntary effort. A combination of pcEmc and fEmc resulted in lowest levels of robotic assistance (Figure 3C). The mean step cycle duration in the presence of stim only reduced from 2.13 s (baseline) to 2.03 s (stim only). However, in the presence of drug the mean step cycle increased to 2.07 s and decreased to 2.00 s with stim+drug. The profile for assistance provided during the course of an average step cycle resulted in a unique pattern of activation of the EKSO compared to when the subject exerted a voluntary effort. These patterns of assistance were consistent with the mean assistance provided. In summary, the absence of pcEmc required higher levels of assistance by the EKSO compared to presence of pcEmc.

### Voluntary control while in supine

**Figure 4:**
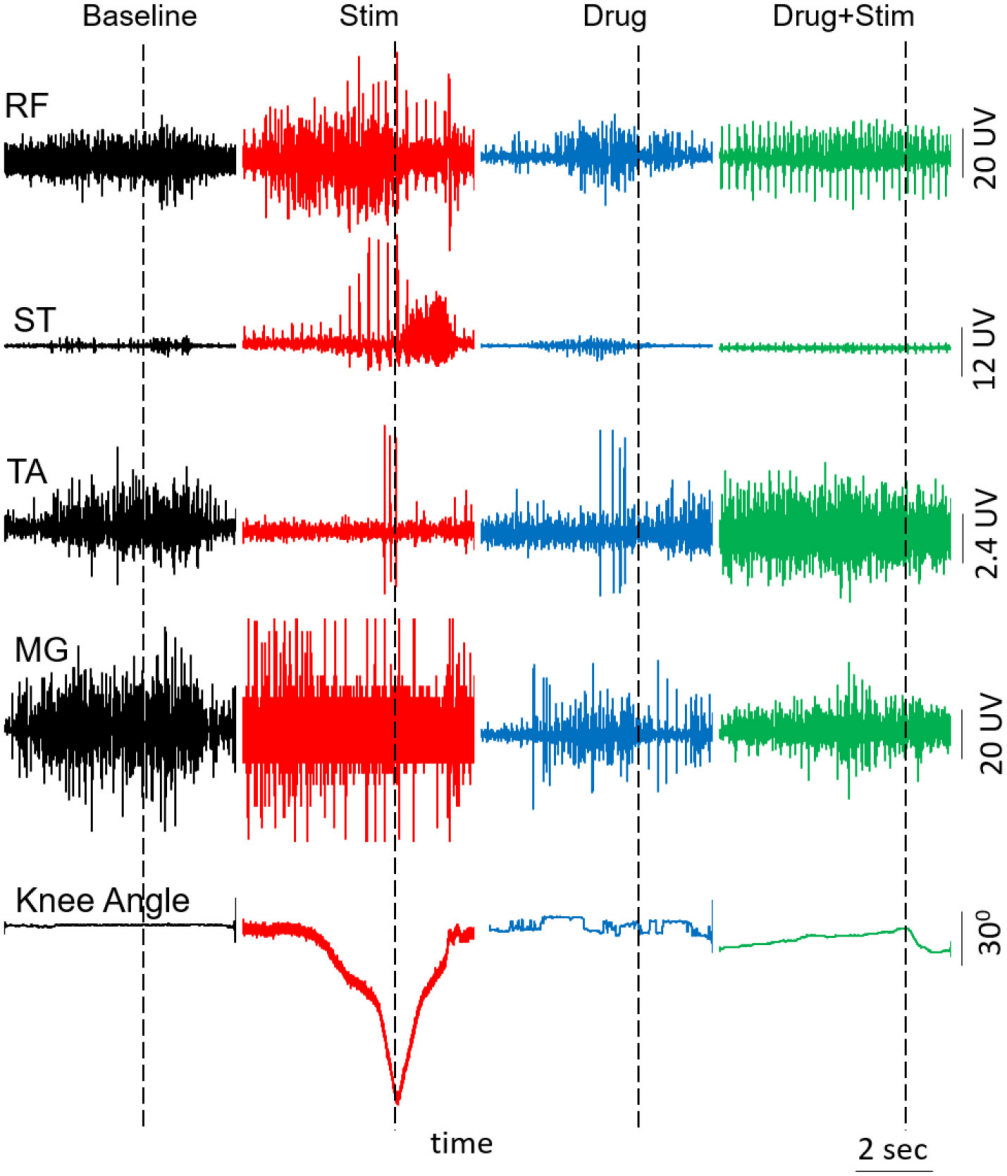
EMG activity and knee angle excursion when attempting voluntary knee flexion when the subject is in a supine position with and without Stim and/or drug. RF, Rectus Femoris; ST, Semitendinosus; TA, Tibialis Anterior; MG, Medial Gastrocnemius.

At the end of each test session, we tested the subject's ability to voluntarily flex his knee with and without pcEmc while lying in a supine position. Without pcEmc, the subject was not capable of flexing his knee but bursting activity was sometimes observed in distal (TA, soleus, and MG) but not in the proximal muscles (VL, RF, and ST). However, in the presence of pcEmc at T11 (30 Hz) and Co1 (5 Hz), the subject was capable of flexing his knee on command (Figure 4) with an alternating bursting pattern corresponding to flexion and extension of proximal muscles with little activity in distal muscles. In the presence of fEmc only, however, the subject was not capable of successfully flexing his knee but generated robust bursting pattern in proximal muscle with little activity in the distal muscles. Further, a combination of pcEmc and fEmc resulted in robust bursting pattern with little change in knee angle.

**Figure 5:**
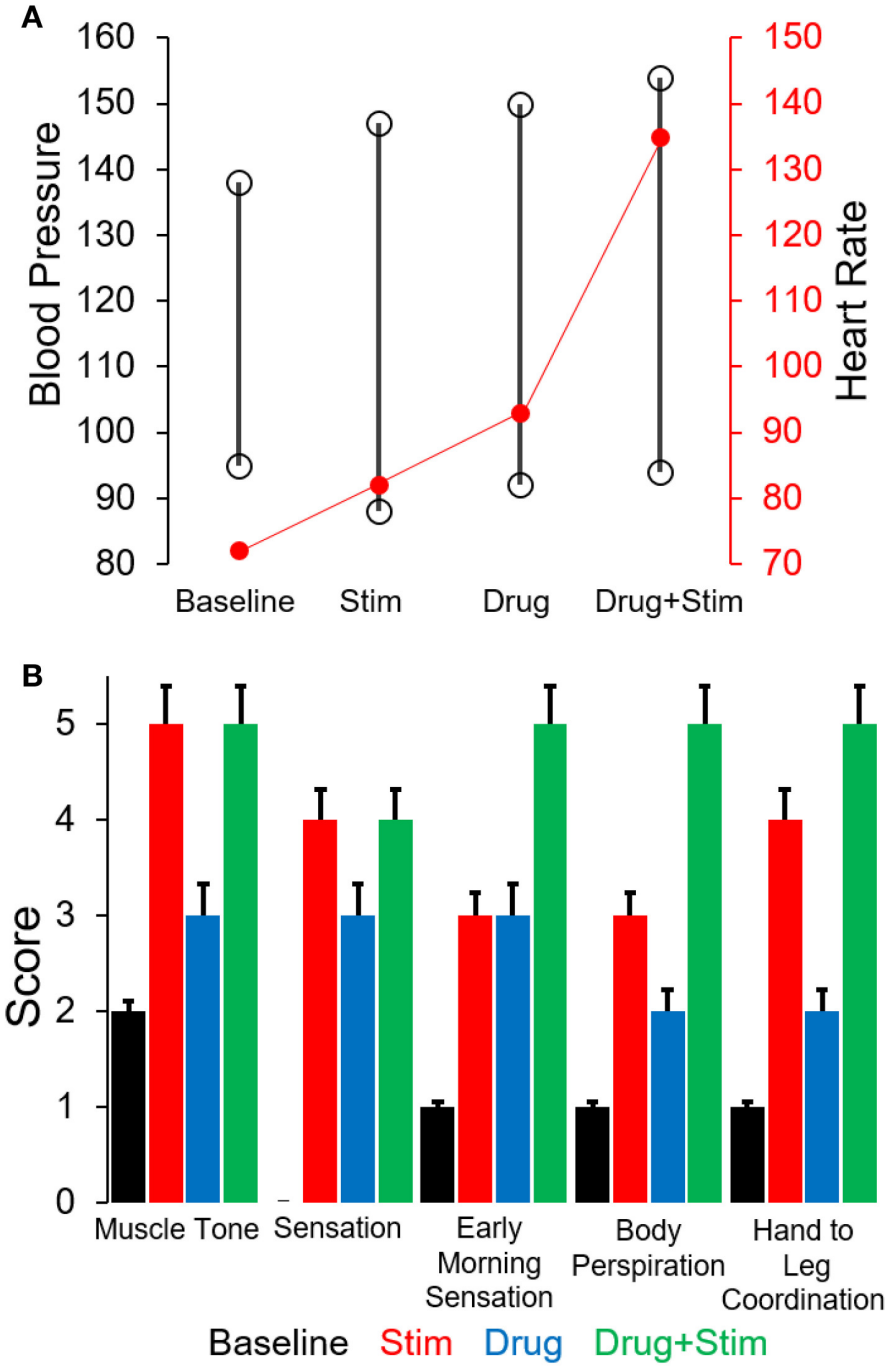
**(A)** Average cardiovascular function (Blood pressure and Heart rate) during the different phases of training. **(B)** Average score (self-scored by subject, 0 = low, 5 = high) on various parameters during the course of various phases of training.

The changes in stepping patterns were accompanied by changes in cardiovascular function (recorded via BP and HR during every training session). The average systolic BP and HR during a single session increased with each phase and was maximum during the combination of pcEmc and fEmc (Figure 5A). Along with changes in EMG patterns and robotic assistance, the subject self-reported changes in muscle tone, sensation, perspiration both during stepping as well as during the rest of the day and hand to leg coordination during stepping. Each of the above variables were higher during either pcEmc or pcEmc+fEmc phases as compared to fEmc only and lowest during baseline (Figure 5B). Further, the trends in the self-reported scores followed the same patterns as the robotic assistance demonstrating the integrated nature of the subject's efforts and the assistance offered by the robot.

## Discussion

Using completely non-invasive interventions, we have demonstrated the ability to neuromodulate the lumbosacral spinal cord to enable function of locomotor networks of a human subject that has been completely paralyzed for more than 4 years. This is consistent with our previous findings with five subjects (Gerasimenko Y. P. et al., 2015). The interventions tested included a combination of non-invasive spinal cord stimulation, pharmacological activation via a monoaminergic agonist (Buspirone), and over-ground weight bearing stepping in an exoskeleton. It has been shown that activation of the lumbar enlargement via transcutaneous electrical stimulation facilitates passive locomotion and robust patterns of EMG activity in lower extremity muscles in SCI patients (Minassian et al., 2007; Hofstoetter et al., 2013, 2015). Recently we have shown that transcutaneous spinal cord neuromodulation (pcEmc) can be used to both initiate oscillatory movement and enable voluntary oscillatory movements in motor complete subjects. These oscillatory movements were further amplified with a combination of pcEmc and fEmc over the course of 18 weeks (Gerasimenko Y. P. et al., 2015).

Stepping in the EKSO Bionics device provides a unique opportunity for subjects with spinal cord injuries to experience over-ground weight bearing stepping (Strausser et al., 2012). However, a unique and perhaps essential advantage of the EKSO Bionics device is the ability to voluntarily engage the lower limbs while stepping, by engaging both supraspinal and spinal networks in a synergistic manner. The EKSO Bionics is capable of functioning in two modes, the maximum assistance mode in which the subject is expected to initiate a step cycle (by shifting his weight to the contralateral side) with the EKSO completing the step cycle with the parameters provided, i.e. step cycle, toe height, swing time etc. while maintaining the decided trajectory. In contrast, while stepping with the variable assistance mode, once the subject initiates a step, the EKSO only provides assistance during the swing phase based on the amount of effort the pilot applies. However, if and when various joints of the active limb falls out of the window of error that is allowed, assistance is provided to ensure the trajectory is maintained and the swing phase is completed as planned. This “assist as needed” paradigm allows the subject to engage the lumbosacral spinal networks to control one limb at a time to complete a swing phase while the contralateral limb goes through a passive stance phase. In addition, pcEmc can modulate the excitability of the spinal neural networks to a state of higher state of excitability that enables re-connectivity of cortical networks to spinal networks projecting to the appropriate motor pools with a relatively high level agonist-antagonistic coordination that supports the generation of standing and stepping.

We have reported similar changes in modulating the physiological state of the lumbosacral spinal cord in four out of the first four subjects diagnosed as AIS A, tested with epidural implants over the L1-S1 spinal levels within weeks of implantation (Harkema et al., 2011; Angeli et al., 2014). The results show continuous recovery in voluntary motor control as a result of daily motor training, but only in the presence of epidural stimulation. In a study of 564 human cadavers with SCI, Kakulas (1999) reported that many of the cadavers had axons within the spinal cord white matter remaining across lesion site even though they were clinically assessed as motor complete. The changes in both locomotor and autonomic systems that we observed in this study provides further evidence that spinal networks of individuals clinically diagnosed at AIS A can be physiologically neuromodulated to a higher and more functional state even with non-invasive neuromodulatory procedures.

Three important aspects of over-ground training in the exoskeleton vs. locomotor training with a body weight support come to light. Firstly, while stepping in an exoskeleton, only one person is needed to spot the subject to avoid falls as compared to 3–4 trainers while stepping on a treadmill. Secondly, while walking in the EKSO Bionics device, the subjects are capable of using their upper bodies, trunk muscles, arms to further enhance their locomotor abilities. Thirdly, over-ground stepping in the EKSO challenges the system to a higher degree compared to stepping with a body-weight support system since the patient has to actively balance the upper body and generate coordinated movements between the arms, trunk and lower limbs. Obviously, the unique feature of the EKSO is that it generates overground stepping kinematics that can be influenced by the subject's effort. The overhead support allowing overground mobility has no means for assisting the limb kinematics. The overhead support devices on a treadmill either imposes uniform kinematics which is markedly non-physiological or relies on multiple skilled therapists. It should be noted that multiple robotic suits are being developed for overground stepping (experimental and FDA approved) with unique sets of built in features (Lebedev and Nicolelis, 2011; del-Ama et al., 2012; Esquenazi et al., 2012; McDaid et al., 2013). Comparing the various features of the different robotic exoskeletons is an important question but that is beyond the scope of the current report.

The present data also demonstrates that the interneuronal spinal networks that normally generates a largely random-stochastic bursting pattern of excitation of the motoneurons can be neuromodulated using a non-invasive mode of stimulation as can occur with epidural stimulation after complete paralysis (Angeli et al., 2014). Convergence of somatosensory and descending motor inputs in combination with spinal cord stimulation and pharmacological activation of interneurons, and perhaps to some degree motoneurons, results in the re-emergence of not only some level of “automated” locomotor movements but also those that can be under more voluntary control (Gerasimenko Y. P. et al., 2015). Once the functionally non-responsive neural networks become electrically responsive, it appears that they can be transformed to a physiological state which is sufficient for them to be re-engaged and even be controlled with the presentation of proprioceptive and cutaneous ensembles of sensory input normally linked to a motor task. It is this new dynamic physiological state which enables it to learn with training.

A more general point to be derived from the present data, when combined with other similar observations (Rejc et al., 2015), questions the validity of several dogmatic concepts, namely, (1) there is limited plasticity after more than a year after a spinal-complete lesion, thus, precluding significant levels of functional recover and (2) with a clinically complete spinal lesion there are no viable cellular components intrinsic to the lesion that can provide a means for supraspinal-spinal re-connectivity. These results in conjunction with our recent report of five subjects with motor complete spinal injuries greater than a year post lesion regaining voluntary influence of lower limb movements within one treatment session with pcEmc raise new and critical questions about the biology of the lesion of a “complete” spinal injury (Gerasimenko Y. P. et al., 2015). The question becomes, to what extent can spared but perhaps functionally incompetent descending axons within the lesion site be transformed to electrically competent networks below the injury using non-invasive painless spinal cord stimulation. If this transformation can be achieved, it appears that descending supraspinal input, proprioception and neuromodulatory inputs projecting to interneuronal networks projecting to different motor pools can generate the appropriate EMG and behavioral response. With long term training, there appears to be an emergence of improved synaptic connections among the spinal interneurons projecting to motoneurons resulting in more “normal stochastic and coordinated bursting patterns” from agonist and antagonistic motor pools compared with dormant networks before training that seems to have formed aberrant functional connections in response to an extensive “denervation” phenomenon within and among spinal networks distal to the lesion.

## Author contributions

PG: Conducted the study, performed data analysis and wrote and edited the manuscript. YG: Conducted the study, wrote and edited the manuscript. SZ: Conducted the study and performed data analysis. AT: Coordinated the study and edited the manuscript. DS: Performed data analysis. DL: Provided medical guidance and edited the manuscript. VE: Conceived the study. Performed data analysis. Wrote and edited the manuscript.

### Conflict of interest statement

VE, YG, DL, and PG, researchers on the study team hold shareholder interest in NeuroRecovery Technologies and hold certain inventorship rights on intellectual property licensed by The Regents of the University of California to NeuroRecovery Technologies and its subsidiaries. The other authors declare that the research was conducted in the absence of any commercial or financial relationships that could be construed as a potential conflict of interest.
